# Odontogenesis-associated phosphoprotein truncation blocks ameloblast transition into maturation in *Odaph*^C41*/C41*^ mice

**DOI:** 10.1038/s41598-020-80912-y

**Published:** 2021-01-13

**Authors:** Tian Liang, Yuanyuan Hu, Kazuhiko Kawasaki, Hong Zhang, Chuhua Zhang, Thomas L. Saunders, James P. Simmer, Jan C.-C. Hu

**Affiliations:** 1 Department of Biologic and Materials Sciences, University of Michigan School of Dentistry, 1210 Eisenhower Place, Ann Arbor, MI 48108 USA; 2 Department of Internal Medicine, Division of Molecular, Medicine and Genetics, University of Michigan Medical School, Ann Arbor, MI 48109 USA; 3 Department of Anthropology, Pennsylvania State University, University Park, PA 16802 USA

**Keywords:** Developmental biology, Evolution, Diseases

## Abstract

Mutations of Odontogenesis-Associated Phosphoprotein (*ODAPH*, OMIM *614829) cause autosomal recessive amelogenesis imperfecta, however, the function of ODAPH during amelogenesis is unknown. Here we characterized normal *Odaph* expression by in situ hybridization, generated *Odaph* truncation mice using CRISPR/Cas9 to replace the TGC codon encoding Cys41 into a TGA translation termination codon, and characterized and compared molar and incisor tooth formation in *Odaph*^+/+^, *Odaph*^+/C41*^, and *Odaph*^C41*/C41*^ mice. We also searched genomes to determine when Odaph first appeared phylogenetically. We determined that tooth development in *Odaph*^+/+^ and *Odaph*^+/C41*^ mice was indistinguishable in all respects, so the condition in mice is inherited in a recessive pattern, as it is in humans. *Odaph* is specifically expressed by ameloblasts starting with the onset of post-secretory transition and continues until mid-maturation. Based upon histological and ultrastructural analyses, we determined that the secretory stage of amelogenesis is not affected in *Odaph*^C41*/C41*^ mice. The enamel layer achieves a normal shape and contour, normal thickness, and normal rod decussation. The fundamental problem in *Odaph*^C41*/C41*^ mice starts during post-secretory transition, which fails to generate maturation stage ameloblasts. At the onset of what should be enamel maturation, a cyst forms that separates flattened ameloblasts from the enamel surface. The maturation stage fails completely.

## Introduction

*C4orf26* (Chromosome 4 open reading frame 26) was unknown to enamel scientists until geneticists found pathogenic variants in the gene that caused autosomal recessive inherited enamel defects in humans^1,2^. The six reported *ODAPH* pathogenic variants included two frameshifts [p.(Cys14Glyfs*21), p.(Val18Cysfs*23)], a splice junction mutation (c.68-2A > T), and three premature termination codons [p.(Cys43*), p.(Arg77*), and p.(Trp106*)]. *C4orf26* was found to be inactivated in toothless placental mammals, so its conservation by natural selection is restricted to its function in teeth^3^. Given this functional specificity, the gene designation was changed from *C4orf26* to *ODAPH* (Odontogenesis-Associated Phosphoprotein, OMIM *614829). Human *ODAPH* encodes a protein of 130 amino acids, including a 23 amino acid signal peptide and a secreted protein of 107 amino acids, which includes 19 prolines, 3 cysteines and multiple serines in context for phosphorylation by the Golgi Casein Kinase complex^4^. This kinase complex includes FAM20A^5^ and FAM20C^6^ and catalyzes functionally critical phosphorylations on three other secreted enamel matrix proteins: enamelin^7,8^, ameloblastin^9^, and amelogenin^10^. In vitro studies showed phosphorylated ODAPH has the capacity to promote nucleation of hydroxyapatite^1^.

Pharyngeal teeth called conodonts were the first tissues to mineralize in chordates/vertebrates, about 515 mya^11^. They served as a feeding apparatus in an eel-shaped, jawless fish^12,13^. Conodonts are analogous, but not homologous, to teeth in jaws^14^. Predation by conodonts may have helped drive the evolution of scales, some of which contained dentin, often topped with a thin hypomineralized layer of enamel or enameloid^15,16^. Scales are homologous to teeth, which evolved by the internalization of scales along with or shortly after the evolution of jaws^17^.

The evolution of bone, dentin, enameloid and enamel involved numerous duplications in SPARCL1 (SPARC Like 1), generating clusters of secretory calcium-binding phosphoprotein (SCPP) genes that mediate biomineralization^18,19^. In the human genome, AMEL (amelogenin) is located on both Xp22.2 and Yp11.2^20^. There are two large SCPP gene clusters on 4q13.3–4q22.1, one encoding 16 proline/glutamine-rich proteins, the other encoding 7 acidic proteins, including SPARCL1^21^. The two clusters are 17 Mb apart. Curiously, *ODAPH*, which is not an SCPP gene, is located in the 17 Mb region between the two clusters (4q21.1).

Dental enamel formation is divided into two main stages: secretory and maturation. During the secretory stage enamel ribbons initiate on the surface of dentin and elongate with continued enamel matrix secretion and assembly to establish the full thickness of the enamel layer. Disturbances to tooth formation that occur during the secretory stage generally reduce the thickness of the enamel layer, causing enamel hypoplasia^22^. Upon completion of the secretory stage, ameloblasts transition into the maturation stage and harden the enamel by growing the thin enamel ribbons deposited during the secretory stage in width and thickness, causing them to interlock^23,24^. Disturbances to tooth formation that occur during the maturation stage generally cause enamel hypomineralization, reducing the hardness of the enamel layer^22^.

To advance our understanding of the role played by ODAPH during tooth formation, we characterized the expression of *Odaph* during mouse tooth development, generated and characterized *Odaph*^C41*^ knockin mice in the C57BL/6N background, and investigated the evolutionary history of the *Odaph* gene during vertebrate evolution. The Cys41* truncation in mouse is homologous to the p.(Cys43*) defect that caused autosomal recessive amelogenesis imperfecta in humans^1^, and should the mutant transcript be expressed, the resulting secreted protein would be very short (18 amino acids) and unlikely to have biological activity.

## Materials and methods

### Regulatory compliance

The animal study protocol was reviewed and approved by the Institutional Animal Care and Use Committee at the University of Michigan and all experiments were performed in accordance with relevant guidelines and regulations.

### Generation of the *Odaph* knockin mouse model

CRISPR/Cas9 technology was used to generate a genetically modified mouse strain carrying the mouse Odontogenesis Associated Phosphoprotein (*Odaph*, formerly *Gm1045*) gene modified by introduction of a translation termination codon (TGA) to replace the cysteine codon (TGC) at position 41. There is only one mRNA transcript variant for mouse *Odaph* (NCBI reference sequence NM_001177577.1). This codon change corresponds to a human *ODAPH* pathogenic variant c.129C > A; p.(Cys43*) that causes autosomal recessive amelogenesis imperfecta^1^.

The following procedures were conducted by the University of Michigan Transgenic Animal Core. A single guide RNA (sgRNA) target and protospacer adjacent motif (PAM) were identified by a search of the mouse *Odaph* coding sequence in Exon 2 (101 bp, NM_001177577.1: nucleotides 65–165 starting from the translation initiation codon) using the CRISPR tool^25^. The sgRNA target to direct Cas9 nuclease cleavage in the Cys41 codon was GAGGAGTGAGTGTGAAGATCTGG (Fig. S1). Ribonucleoprotein complexes of 30 ng/µL sgRNA + 30 ng/µL Cas9 protein were injected into fertilized mouse eggs by pronuclear microinjection to test for sgRNA induced Cas9 mediated chromosome breaks. Eggs were permitted to develop to the 64-cell blastocyst stage in vitro and then DNA was extracted from individual blastocysts for analysis. A PCR fragment spanning the expected Cas9 cut site was amplified and the product characterized by DNA sequence analysis. Small insertions/deletions (indels) caused by nonhomologous end joining repair of Cas9 mediated chromosome breaks were detected by the presence of multiple overlapping templates in the Sanger sequencing reaction. An ultramer DNA oligonucleotide donor was obtained from IDTDNA.com: TGGTTGGTGGTAACTACAGCAGAAGGACAAGATGTAGTCACCCCTCCTGGCGGCTCACAAAATAACGCAAAGCCTACcGAtTGaCAGATtTTtACcCTgACTCCTCCGCCCACCACAAGGAATCTGGTAACAAGGGCCCAGCCCATCCCAAGGACACCCACGTTTTCTTTTCCLower case letters indicate changes introduced to block sgRNA binding and Cas9 induced chromosome breaks after the oligonucleotide was incorporated into the chromosome by homology directed repair. These sequence changes were strategically introduced into the *Odaph* allele to eliminate a *Bgl*II restriction site and to generate a mutant-allele-specific primer annealing site to simplify genotyping. In the fertilized C57BL/6J eggs, homology directed repair of the chromosome replaced the homologous wild-type sequence with that of the symmetrical oligonucleotide donor. Fertilized eggs were produced by mating superovulated C57BL/6J female mice mated with C57BL/6J males (Jackson Laboratory Stock 000664). The genome editing reagent mixture for microinjection contained 30 ng/µL sgRNA + eSpCas9(1.1) protein (50 ng/µL) + oligonucleotide donor 10 ng/µL. After the microinjection, surviving eggs were transferred to B6D2F1 pseudopregnant recipients (Jackson Laboratory Stock 100006). DNA samples were collected for genotype analysis from the pups born after embryo transfer.

### Germline transmission

Genotyping assays identified three female and two male G0 founder mice that carried the *Odaph* p.Cys41* mutation. The G0 founder mice were crossed with C57BL/6 N (Charles River, strain code 027) for two generations to dilute any possible off-target effect of the CRISPR/Cas9 gene editing and to generate offspring for characterization. All G0 founders demonstrated germline transmission. Neither the founders nor their offspring had observable changes in their behavior and no apparent physical defects from neonatal stage to young adulthood. Genotyping and sequence validation were conducted as described in Fig. S2.

### *Odaph*^C41*^ genotyping and validation

DNA collected from tail biopsies was amplified using *Odaph*-specific primers F: 5′ ATTCCTCCATAAAATCACATTTGTGCTGA and R: 5′-AATGTGTAATCCAAACTCCTTGTTGTTGA that generated a 996 bp amplification product in both the wild-type and mutant mice. The PCR conditions were 94 °C for 2 min, then 35 cycles of [94 °C for 30 s, 59 °C for 30 s, 72 °C for 30 s] followed by 72 °C for 60 s and then hold at 4 °C. Each PCR reaction contained 10 µL of Platinum Hot Start PCR Master Mix (2×) (Invitrogen, Carlsbad, CA, USA), 1 µL of 10 µM Primer mix, 3 µL of DNA (40–50 ng/µL) template and 6 µL distilled water. The reactions were run using a GeneAmp PCR System 9700 (Applied Biosystems, Foster City, CA, USA). The amplification products were restricted with *Bgl*II, which only cleaves the WT amplicon as a *Bgl*II site was eliminated by the CRISPR design. Each reaction contained 10 µL of PCR products (~ 0.2 µg), 2.5 µL of NEB buffer 3.1 (10×) and 0.6 µL of NEB *Bgl*II (New England Biolabs, Rowley, MA, USA) and raised to 25 µL with distilled water. The reactions were incubated at 37 °C for 30 min and loaded on 1.5% agarose gel. The *Odaph*^C41*^ amplicon was 996 bp; the restricted *Odaph*^+/+^ amplicon was 560 and 436 bp (Fig. S2A). The *Odaph*^C41*/C41*^ amplicon was characterized by DNA sequencing and showed no differences with the wild-type except those intended, which are shown in the DNA sequence chromatograms (Fig. S2B). All coding exons and exon–intron junctions of F1 mice were confirmed by Sanger sequencing. F1 mice with correctly targeted sequences were bred with B6 to generate F2 for colony expansion.

RNA was isolated from enamel organ epithelia (EOE) dissected from *Odaph*^C41*/C41*^ and *Odaph*^+/+^ first molars at D5 (secretory stage) and D11 (maturation stage) using a Dynabeads mRNA Direct Purification Kit (Invitrogen, Carlsbad, CA, USA), converted to cDNA by reverse transcription, PCR amplified using Platinum Hot Start PCR Master Mix (2×) (Invitrogen) and visualized on a 1.5% agarose gel. *Odaph*-specific primers F: 5′- GTGAGTGCTCAGGGCAGAA; R: 5′-CGGTCTCATTAAAATGTCCTTCA generated a 753 bp amplification product in both the wild-type and mutant. The PCR conditions were 94 °C for 2 min, then 28 cycles of [94 °C for 30 s, 58 °C for 30 s] followed by 72 °C for 60 s. A similar amplification was run using *Gapdh*-specific primers F: 5′-AGGCCGGTGCTGAGTATGTC; R: 5′-TGCCTGCTTCACCACCTTCT that generated a 530 bp amplification product.

### Dissecting microscopy

Seven-week-old *Odaph*^+/+^, *Odaph*^+/C41*^, and *Odaph*^C41*/C41*^ mice were lightly anesthetized with isoflurane and their frontal facial images were taken using a dissection microscope. At least three samples from each genotype were assessed. A separate group of mice were deeply anesthetized with isoflurane and perfused with 1 × phosphate buffered saline (PBS) for 10 min. Their mandibles were dissected and denuded of soft tissues, post-fixed by immersion in 4% paraformaldehyde (PFA) overnight, and rinsed with PBS three times, for 5 min each. The teeth were cleaned with 1% bleach (sodium hypochlorite), rinsed with PBS, air dried, displayed on the Nikon SMZ1000 dissection microscope, and photographed using a Nikon DXM1200 digital camera, as described previously^26^.

### In situ hybridization

Mandibles containing developing incisors were harvested from wild-type C57BL/6 N mice at 7-weeks, formalin-fixed, decalcified in a solution of 150 mM NaCl/10% acetic acid, paraffin embedded, microtome sectioned, and deparaffinized in xylene. An antisense *Odaph* RNA probe for RNAscope in situ hybridization was designed and produced by Advanced Cell Diagnostics (Newark, CA, USA). RNAscope 2.5 Assay with RED HD Detection Reagent (Advanced Cell Diagnostics, Inc. Newark, CA, USA) was performed for in situ hybridization, following user manual 322452 (FFPE sample preparation and pretreatment) and 322360 (RNAscope 2.5 HD Detection Reagent – RED user manual) provided by the manufacturer, as described previously^27^. The following probes were used: (1) Mm-Odaph (Cat #576061, targeting NM_01177577.1, nt 2-743); (2) Mm-Klk4 (Cat #483451, targeting NM_019928.1, nt 235-1228); and (3) Neg Ctrl Probe_dapB (Cat #310043). Photographs were taken using a Nikon Eclipse TE300 microscope equipped with a Nikon DXM1200 digital camera.

### Histological analysis

Heads of *Odaph*^+/+^, *Odaph*^+/C41*^, and *Odaph*^C41*/C41*^ mice at postnatal days 5, 8 and 12 were harvested and the mandibles were dissected from the heads. Samples were fixed in 4% paraformaldehyde in diethyl pyrocarbonate (DEPC)-treated phosphate buffered saline (PBS; 137 mM NaCl, 2.7 mM KCl, and 11.9 mM phosphates) at 4 °C overnight (~ 18 h). The samples were decalcified in DEPC-treated 4.13% disodium ethylenediaminetetraacetic acid (EDTA, pH 7.4) at 4 °C with agitation, with the EDTA solution changed every 3 days for 5 to 12 days, depending on the ages. The samples were then dehydrated and embedded in paraffin. Mesial-distal sections (5 µm) were prepared for histological analysis. Hematoxylin and Eosin (H&E) staining was described previously^26^. Photos were taken using a Nikon Eclipse TE300 microscope, and photographed using a Nikon DXM1200 digital camera.

### Backscattered scanning electron microscopy (bSEM)

Whole surface scanning was performed on 7-week-old molars of *Odaph*^+/+^, *Odaph*^+/C41*^, and *Odaph*^C41*/C41*^ hemi-mandibles. Hemi-mandibles were submerged in 4% PFA overnight and on the following day were carefully dissected of soft tissues, submerged in 1% NaClO for 20 min, rinsed, dehydrated using an acetone series (30, 50, 70, 80, 90, and 100%) and air dried. The hemi-mandibles were mounted on metallic stubs using conductive carbon cement, and imaged using a Joel JSM-7800FLV field-emission scanning electron microscope operating at an accelerating voltage of 15 kV in the backscatter mode at the University of Michigan Robert B. Mitchell Electron Microbeam Analysis Lab (EMAL, Ann Arbor, MI, USA).

Mouse incisors and molars were prepared for bSEM characterization following an optimized protocol^28^. Seven‐week‐old *Odaph*^+/+^, *Odaph*^+/C41*^, and *Odaph*^C41*/C41*^ mice were anesthetized with isoflurane, perfused with 4% PFA, dissected free of soft tissue, dehydrated with an acetone series (30, 50, 70, 80, 90, and 100%), embedded in epoxy, and cross sectioned at 1 mm increments along their lengths as described previously^29,30^. Wild-type incisor cross sections at Level 8 align with the buccal crest of alveolar bone and show fully formed enamel that has not yet erupted into the oral cavity. The polished cut surface was coated with carbon to increase conductivity and examined at × 5000 magnification in Joel 7800 (JEOL USA, Inc., Peabody, MA, USA) using the backscatter mode at a beam current of 20 kV and 10 nA. Images were captured at a working distance of 10 mm, with minor adjustments to focus. Selected images were normalized to have the same mean grayscale intensities for mineralized dentin using ImageJ (http://rsb.info.nih.gov/ij/), so that the grayscale of bSEM images from different images would most accurately be compared for degree of mineralization (whiter = higher mineralization).

### Identification of *Odaph* orthologs

*Odaph* orthologs were identified in the window of human *Odaph* at the UCSC genome browser (http://genome.ucsc.edu/index.html) using the conservation track. For non-eutherian orthologs, which show limited sequence identities at the nucleotide level, genomic regions syntenic to human *Odaph* was investigated (downstream of *Cdckl2*), and intron spanning RNA-seq readings were searched for the *Odaph* ortholog at either NCBI genome browser (https://www.ncbi.nlm.nih.gov/) or Ensemble genome bowser (http://useast.ensembl.org/index.html).

## Results

### The *Odaph*^C41*^ dental phenotype

**Figure 1:**
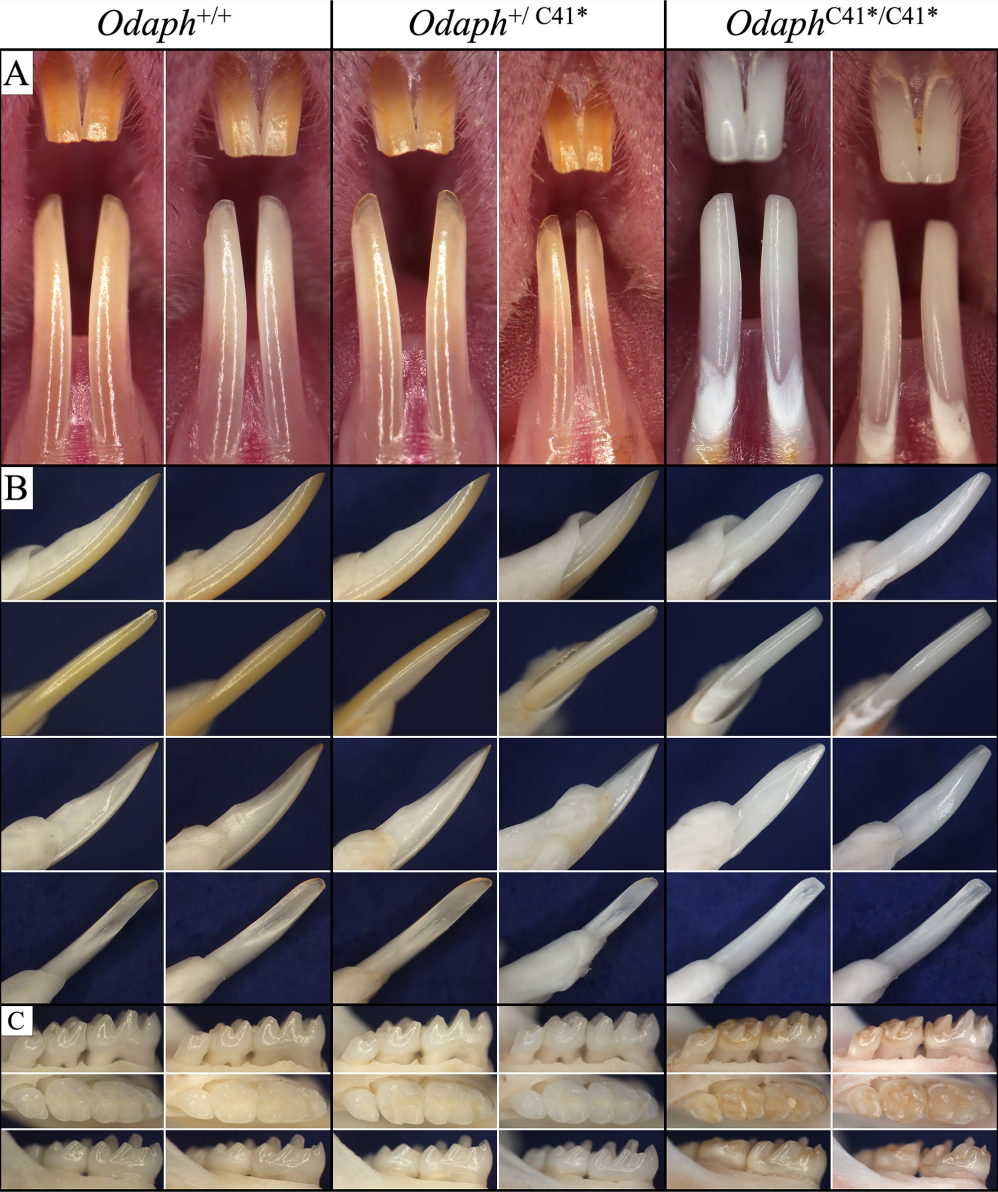
Appearance of *Odaph*^+/+^, *Odaph*^+/C41*^, and *Odaph*^C41*/C41*^ Incisors and Molars at 7-weeks. There were no appreciable differences in dental phenotype observed between *Odaph*^+/+^ and *Odaph*^+/C41*^ mice. Tooth size and morphology were similar in the three genotypes. (**A**) Frontal view of maxillary and mandibular incisors. The *Odaph*^C41*/C41*^ incisors appeared to be severely hypomineralized. Their incisors were chalky white and the enamel had abraded from dentin surface down to the cervical region. (**B**) Mesial, labial, distal, and lingual (lower right) views of the mandibular incisors. The *Odaph*^C41*/C41*^ incisal edge is flat and appears to be shorter than the wild-type due to attrition. (**C**) Buccal, Occlusal, and Lingual views of mandibular molars. No differences in alveolar bone level were observed among the three genotypes. The *Odaph*^C41*/C41*^ molars were discolored and had undergone significant attrition.

Using the CRISPR/Cas9 system we generated *Odaph*^C41*^ mice in the C57BL/6J background by converting the codon for Cys41 into a translation termination codon. The founders were back-crossed with C57BL/6N wild-type mice for two generations to remove potential off-target effects of the CRISPER/Cas9 editing process. Based upon RT-PCR analyses, the *Odaph*^C41*^ allele appeared to be expressed in both the secretory and maturation stages of amelogenesis, and did not appear to undergo nonsense mediated decay (Fig. S2C). If expressed, the mutant allele would secrete an 18 amino acid peptide: QDVVTPPGGSQNNAKPTD*, reduced from the 103 amino acids of the wild-type. The dental phenotype was first inspected under a dissecting microscope (Fig. 1). The homozygous *Odaph*^C41*/C41*^ mice could be readily distinguished from their wild-type (*Odaph*^+/+^) and heterozygous (*Odaph*^+/C41*^) littermates because of their apparent enamel defects. The incisor enamel was chalky-white and underwent rapid attrition (Fig. 1A,B), despite the animals being fed soft chow. The molar enamel showed a brown discoloration and also underwent rapid attrition (Fig. 1C). The heterozygous *Odaph*^+/C41*^ mice appeared to be normal in all respects.

### Timing of *Odaph* expression and pathology

**Figure 2:**
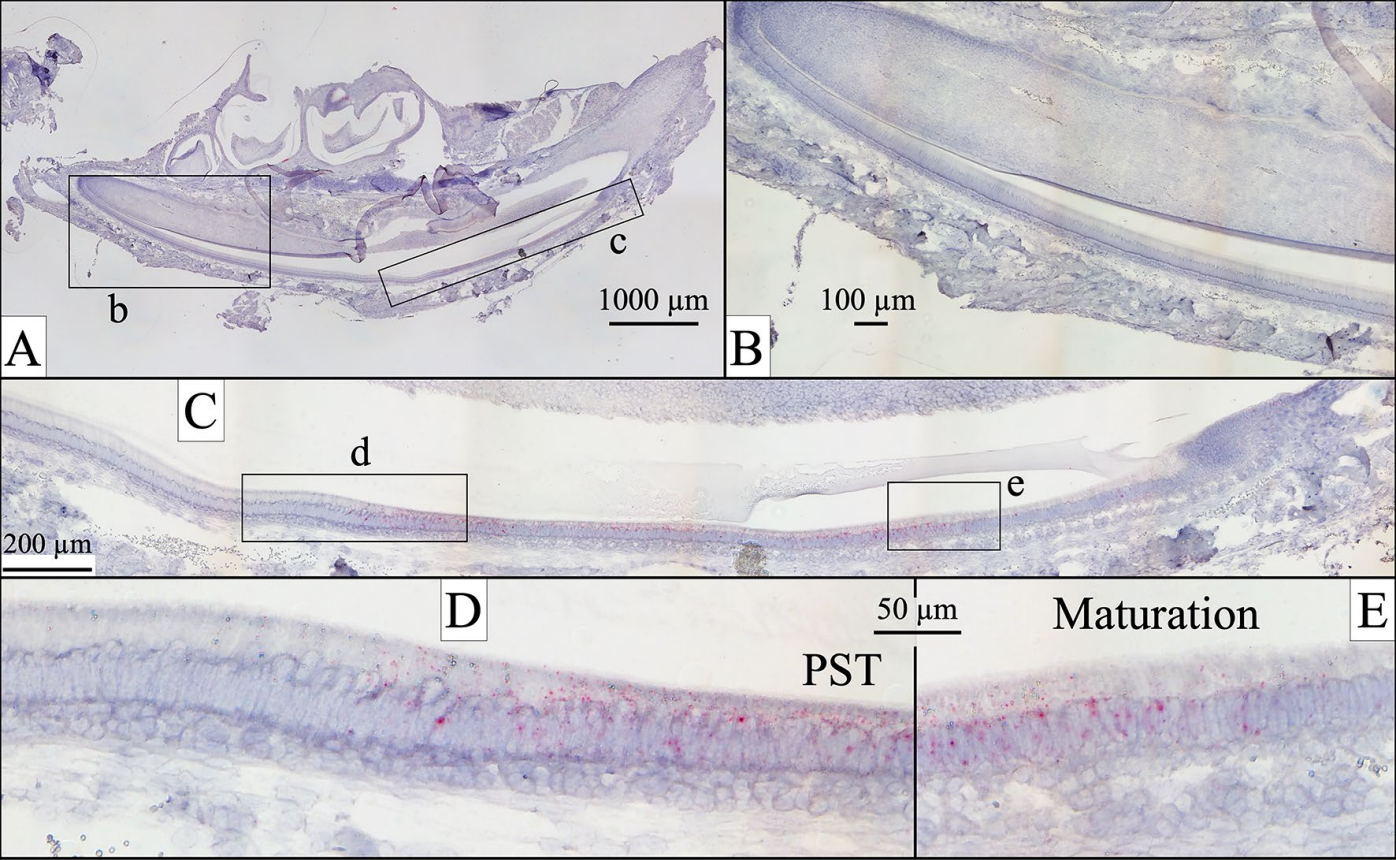
*Odaph* Expression in *Odaph*^+/+^ Day 12 Mandibular Incisors. In the continuously growing mandibular incisor enamel formation progressively advances from basal (left) to incisal (right). (**A**) Sagittal section of a D12 mouse hemimandible. Boxes show the locations of the higher magnification views in (**B**) and (**C**). (**B**) *Odaph* expression was absent or trace in presecretory and secretory stage ameloblasts. (**C**) Late secretory, post-secretory transition (PST) and early maturation stages of amelogenesis. Boxes show the locations of the higher magnification views in (**D**) and (**E**) *Odaph* expression was low, but could be detected from the middle of box (**D**) to nearly the end of box (**E**). (**D**) On the left the ameloblasts were ending the secretory stage. Approximately midway through image (**D**), they entered post-secretory transition (PST) where the ameloblasts normally shorten while converting to ruffle-ended maturation stage ameloblasts. Note that *Odaph* expression is observed at the onset of PST. (**E**). Early maturation ameloblasts were positive for *Odaph* expression. No significant *Odaph* signal was detected in odontoblasts.

**Figure 3:**
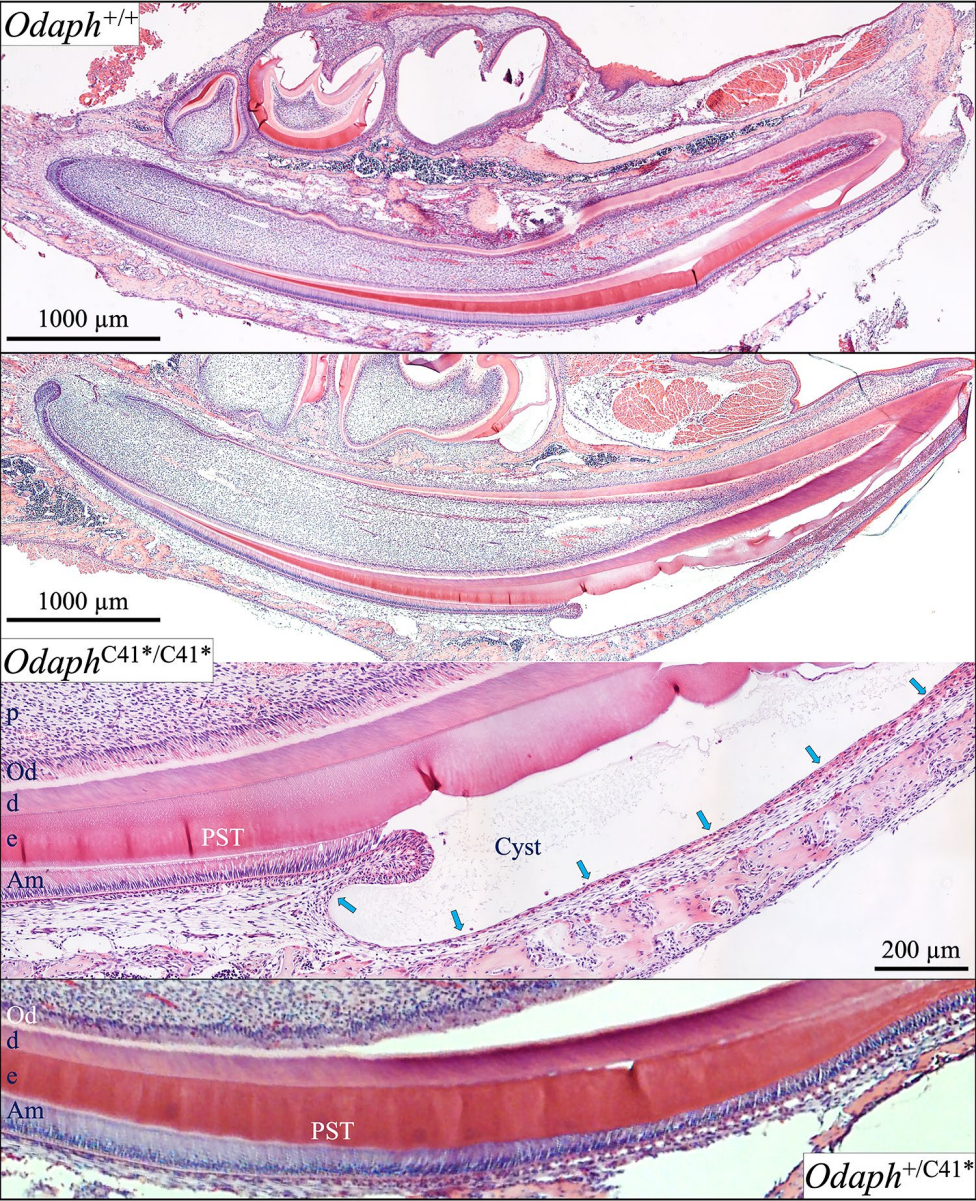
*Odaph*^+/+^, *Odaph*^+/C41*^, and *Odaph*^C41*/C41*^ Mandibular Incisor Histology at D12. Incisor sagittal sections provide histological information on all stages of enamel formation, including differentiating, presecretory, secretory, transition and maturation ameloblasts. The *Odaph*^+/C41*^ incisors (bottom) resembled those of the wild-type (top). The *Odaph*^C41*/C41*^ incisors appeared to be normal until late in post-secretory transition (PST) when ameloblasts abruptly reduced in height and flattened (arrows) at the onset of the maturation when a cyst appeared between the ameloblasts and the enamel surface. Key: p: pulp, Od: odontoblasts, d: dentin, e: enamel, Am: ameloblasts, PST: post secretory transition stage of amelogenesis.

To discern the timing of normal *Odaph* expression and the onset of enamel malformations in *Odaph*^C41*/C41*^ mice, we conducted in situ hybridization studies on sagittally sectioned D12 hemimandibles using custom *Odaph* riboprobes. Developing mandibular incisors display all stages of amelogenesis in a linear array and gave a clear picture of the timing of *Odaph* expression (Fig. 2). Although the positive signal was weak relative to what we have observed for other enamel proteins, it was specific for ameloblasts and showed a clear onset in post-secretory transition and terminated in mid-maturation. No other cells besides ameloblasts were positive. We compared the histology of D12 mandibular incisors from *Odaph*^+/+^ and *Odaph*^C41*/C41*^ mice (Fig. 3). The *Odaph*^C41*/C41*^ incisors appeared to be normal until midway through post-secretory transition. At what should have been the onset of enamel maturation, a cyst formed that separated the ameloblasts from the underlying enamel and flattened the ameloblasts into squamous cells. These events must eliminate the entire maturation process of removing residual enamel protein and hardening the enamel layer by thickening the thin enamel crystals deposited previously during the secretory stage.

**Figure 4:**
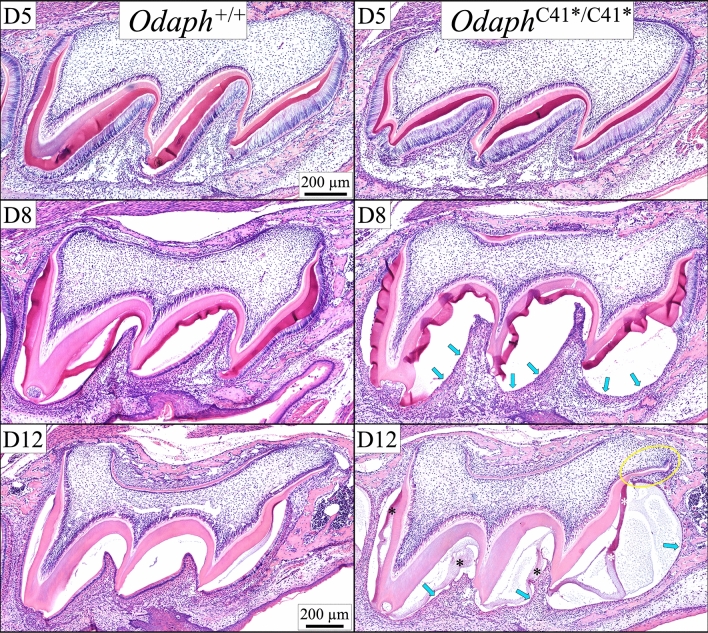
*Odaph*^+/+^ and *Odaph*^C41*/C41*^ Maxillary First Molar Histology at D5, D8 and D12. Day 5 first molar ameloblasts have not yet entered the maturation stage. The histology appearances of *Odaph*^+/+^ and *Odaph*^C41*/C41*^ secretory stage ameloblasts in D5 molars were similar to each other and the degree of enamel and dentin matrix deposition was comparable. Day 8 wild-type first molar ameloblasts are predominantly in the maturation stage. The maturation stage ameloblasts in D8 *Odaph*^C41*/C41*^ molars were flattened along the cuspal slopes (arrows) and the enamel space was expanded, due to apparent cyst formation. Dentin and root development appeared to be normal. On Day 12 molars, polarized late-maturation stage ameloblasts covered the *Odaph*^+/+^ molars, whereas only flattened epithelial cells (arrows) covered the same area in the *Odaph*^C41*/C41*^ molars, and residual enamel matrix (*) was still apparent in the enamel space. Mesial root development was distorted (yellow circle) presumably due to the expanding cystic space at the cervical margin. Dentin, root development, and alveolar bone appeared to be normal.

We compared the histology of developing maxillary first molars from *Odaph*^+/+^ and *Odaph*^C41*/C41*^ mice at successive stages of development: on D5 (secretory stage), D8 (transition and early maturation) and D12 (late maturation) (Fig. 4). The results were consistent with those for the incisors. Ameloblasts, odontoblasts, dentin and enamel in Day 5 *Odaph*^C41*/C41*^ maxillary molars were indistinguishable from those in the wild-type. On Day 8 cysts were formed on the cusp slopes that expanded the enamel space and flattened the ameloblasts. Root and dentin development appeared to be normal. On D12, polarized maturation ameloblasts were neatly arrayed over the enamel space in the wild-type and only small amounts of residual protein remained in the enamel. In the *Odaph*^C41*/C41*^ molars, flattened epithelial cells lined a cyst-covered enamel space and abundant residual enamel protein remained in the enamel. Although root development progressed similarly in both groups, the mesial root of the *Odaph*^C41*/C41*^ molar was distorted, apparently by pressure from the cyst overlying the adjacent enamel. No apparent pathosis was observed in the surrounding alveolar bone or oral epithelium.

**Figure 5:**
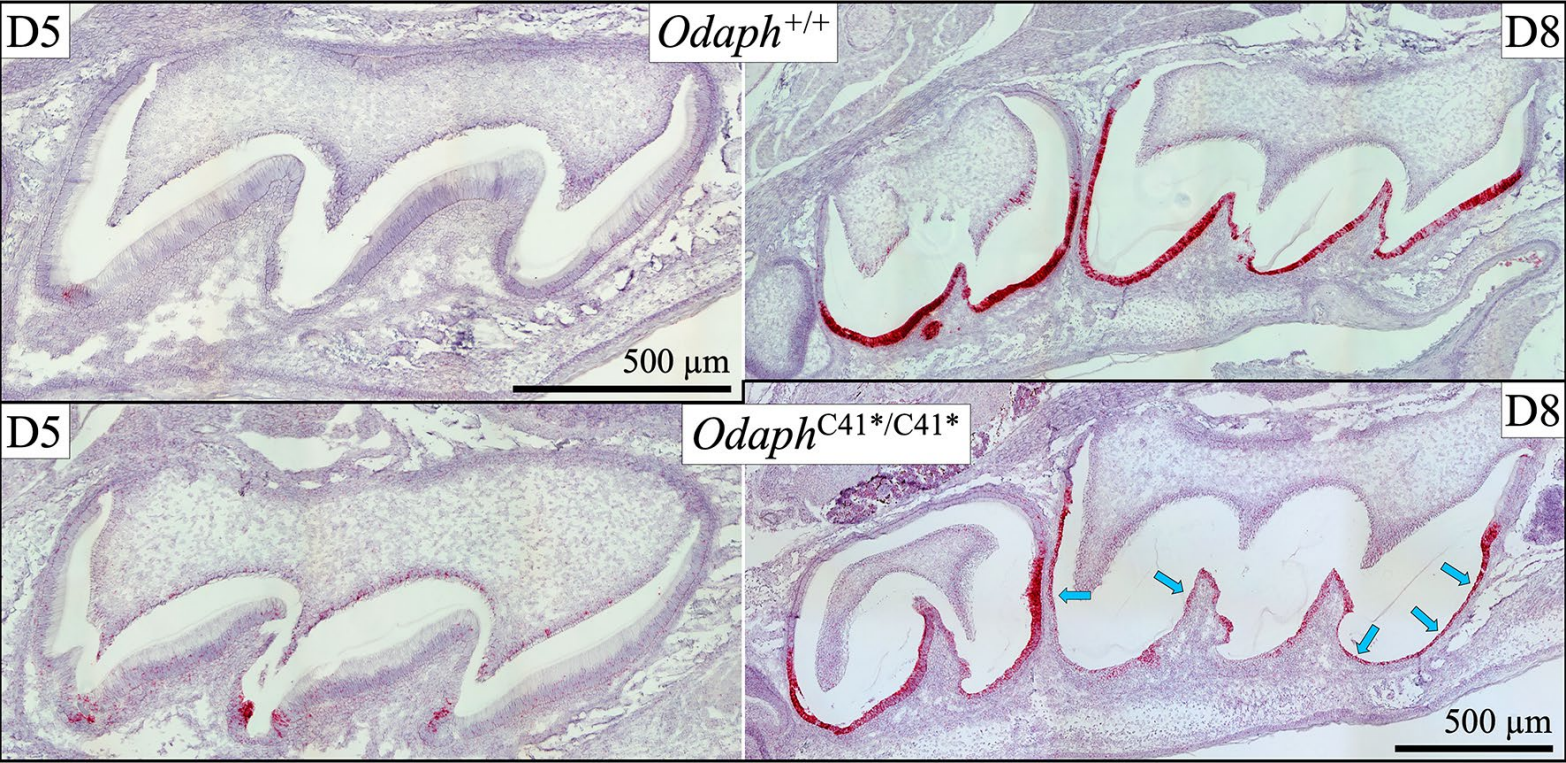
*Klk4* Expression in *Odaph*^+/+^ and *Odaph*^C41*/C41*^ D5 and D8 Maxillary First Molars. On Day 5, positive in situ hybridization for *Klk4* expression was observed in some odontoblasts and in the enamel free zone near the cusp tips of both *Odaph*^C41*/C41*^ and *Odaph*^+/+^ maxillary first molars. On Day 8, after enamel development had progressed into the early maturation stage, positive *Klk4* mRNA signal was specifically observed in transition and maturation ameloblasts of wild-type first and second molars, with spotty signal in odontoblasts. A similar pattern was observed in D8 *Odaph*^C41*/C41*^ maxillary molars but with decreased intensity (arrows) in the flattened ameloblasts lining the expanded, cyst-like enamel space. This *Klk4* expression pattern, although diminished relative to the wild-type, supports the interpretation that the flattened epithelial cells lining the cyst were ameloblasts.

To better visualize the ameloblasts during transition and early maturation, we performed in situ hybridization of *Klk4* mRNA on wild-type and *Odaph*^C41*/C41*^ molars, because *Klk4* has a pattern of expression that closely matches that of *Odaph*^31–33^, with an identical onset in post-secretory transition (Fig. 5). Specific *Klk4* expression was evident in the flattened cells lining the cysts covering maturation enamel in *Odaph*^C41*/C41*^ molars, supporting the interpretation that the ameloblasts had not died, but had flattened and lined the cysts. The histology strongly suggested that ODAPH was essential for a normal transition from secretory to maturation ameloblasts and potentially for the adhesion of maturation ameloblasts to the enamel surface. In *Odaph*^C41*/C41*^ mice this attachment fails and fluid accumulates in the space between ameloblasts and the enamel surface.

### Backscattered scanning electron microscopy

**Figure 6:**
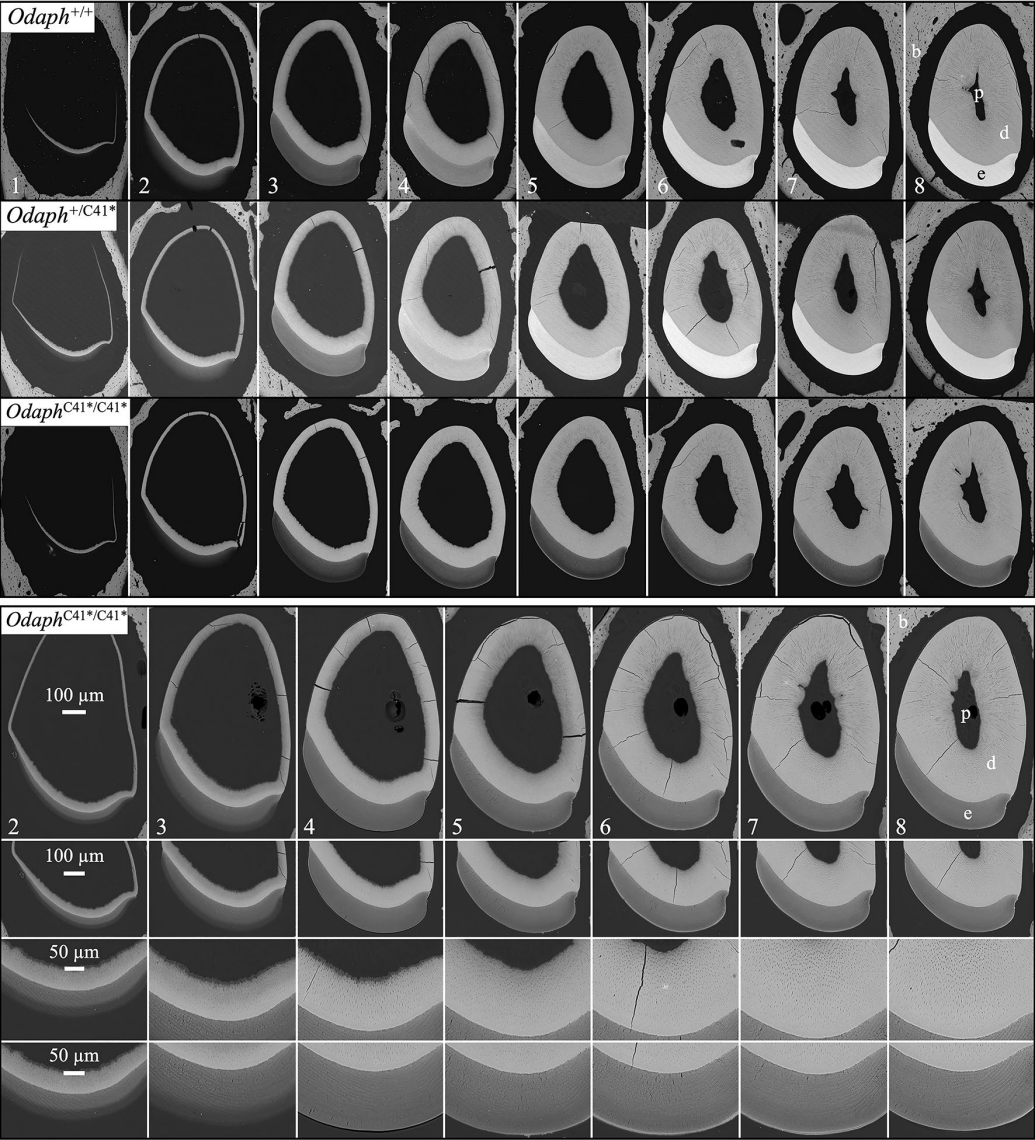
bSEM of 7-wk *Odaph*^+/+^, *Odaph*^+/C41*^, and *Odaph*^C41*/C41*^ Mouse Mandibular Incisor Cross-Sections. These panels show 1-mm (basal, left; incisal, right) incremental cross-sections of 7-wk mandibular incisors taken from *Odaph*^+/+^, *Odaph*^+/C41*^, and *Odaph*^C41*/C41*^ mice. Top: Comparison of the 3 genotypes from levels 1 to 8 showed that the early progress of dentin and enamel deposition is similar in all three genotypes, with the enamel layer having achieved its final contour and thickness by level 3. After level 3 the striking finding was the failure of the *Odaph*^C41*/C41*^ enamel layer to increase in density, as occurred in the *Odaph*^+/+^ and *Odaph*^+/C41*^ incisors. Bottom: High magnification views of *Odaph*^C41*/C41*^ sections showed normal dentin and dentin tubules and enamel rod decussation patterns and final enamel thickness. The only deficiency was the dramatic lack of enamel maturation in the *Odaph*^C41*/C41*^ incisors. The *Odaph*^C41*/C41*^ enamel surface was the only part of the enamel layer that increased in density. Key: b: bone, p: pulp, d: dentin, e: enamel.

bSEM produces an image like a dental radiograph, with more highly mineralized materials being whiter, and less mineralized materials being grayer. bSEM images of 7-week-old mandibular incisor cross-sections taken at 1 mm increments are a standard means of visualizing the progress of tooth mineralization^30^. Level 1 through Level 3 corresponds to the secretory stage, while levels 4 through 8 show enamel maturation (Fig. 6). Mineralization of dentin is the same in *Odaph*^+/+^, *Odaph*^+/C41*^, and Odaph^C41*/C41*^ mice at all stages (Fig. 6, top). In addition, the enamel layer achieves its full thickness and normal contour by Level 3 in all three genotypes. This is very different from the bSEM results when a gene necessary for the secretory stage is affected and the enamel layer fails to expand significantly, as in amelogenin^34,35^, enamelin^36^, ameloblastin^37^, and matrix metallopeptidase 20 (MMP20)^28,38^ null mice.

**Figure 7:**
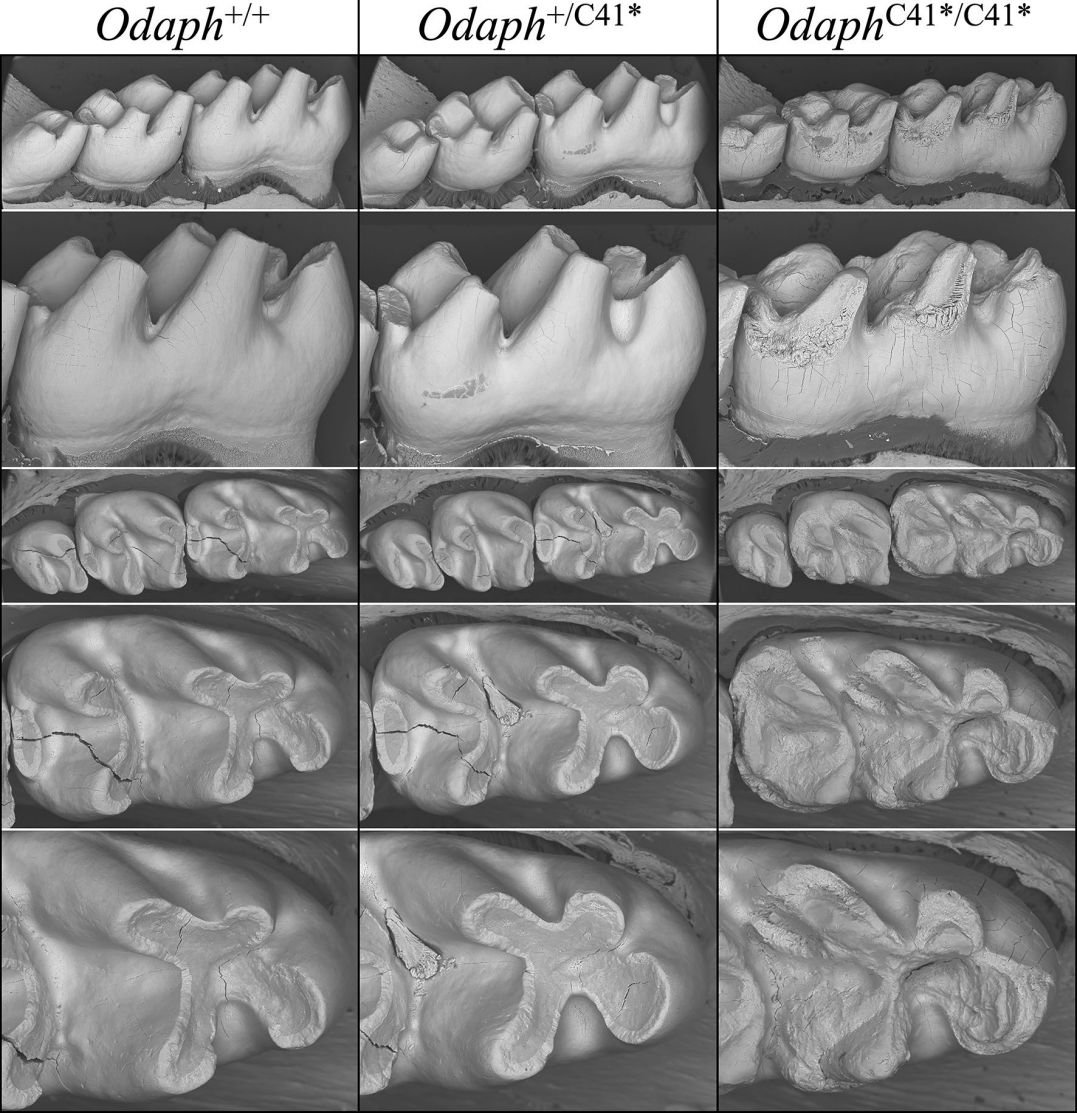
bSEMs of 7-wk *Odaph*^+/+^, *Odaph*^+/C41*^, and *Odaph*^C41*/C41*^ Mouse Mandibular First Molars. Lingual (top 2 rows) and occlusal (bottom 3 rows) views of 7-wk mandibular molars. No differences in attrition were observed between *Odaph*^+/+^ and *Odaph*^+/C41*^ molars. The *Odaph*^C41*/C41*^ molars showed extensive attrition. Like the incisors, the molar enamel seemed to reach full thickness but was soft and underwent attrition following eruption.

The one major difference among the three genotypes is that from Level 4 to Level 8 the enamel layer in *Odaph*^C41*/C41*^ incisors fails to increase in mineral density and remains at the same gray scale, suggesting a complete absence of enamel maturation. This pattern is similar, but somewhat more severe in terms of the reduction in mineral density observed in *Odaph*^C41*/C41*^ maturation stage enamel compared to other mouse knockouts with defects specific to the maturation stage (Fig. S3), such as *Klk4*^39^ and *Wdr72*^40^. In addition to the *Odaph*^C41*/C41*^ enamel layer achieving full thickness (but not density), the decussation pattern of enamel rods in the enamel layer was unaffected (Fig. 6, bottom). Enamel defects corresponding to a specific failure of enamel maturation were also observed in the bSEM images of mandibular molars at 7-wks (Fig. 7). The enamel crowns in the molars of all three genotypes showed normal shape and contour (indicative of a normal secretory stage), but the enamel of *Odaph*^C41*/C41*^ was softer and underwent significant attrition compared to the wild-type and *Odaph*^+/C41*^ molars.

## Discussion

*Odaph* expression detected by in situ hybridization was very low in developing mouse teeth. A second riboprobe was ordered and gave the same result. Although the signal was low, it was specific for ameloblasts starting at the onset of ameloblast transition and continuing throughout early maturation stage. As it turned out, the dental phenotype observed in the *Odaph*^C41*/C41*^ knockin mice was entirely consistent with a disturbance in tooth development occurring during the narrow window when *Odaph* was shown to be expressed by in situ hybridization.

A simple inspection of the incisors could distinguish between mice heterozygous (*Odaph*^+/C41*^) or homozygous (*Odaph*^C41*/C41*^) for the *Odaph* truncation. Closer histologic examination of developing molars and incisors showed that secretory stage ameloblasts and the forming enamel layer appeared normal in all three genotypes. bSEM analyses showed the *Odaph*^+/C41*^ enamel layer grew to normal thickness and contour, and achieved the same high degree of mineralization and resistance to attrition as the enamel of wild-type mice. The pattern of inheritance of *Odaph* Cys41* mutations in mice is autosomal recessive. This is consistent with the pattern of inheritance in humans and suggests that if the truncated protein had been expressed, it caused no detectable pathosis. Expression of half the normal amount of ODAPH was sufficient for normal amelogenesis in *Odaph*^+/C41*^ mice.

The histology of *Odaph*^C41*/C41*^ molars and incisors showed normal differentiating and secretory stage ameloblasts, but a dramatic failure of ameloblasts to transition normally into the maturation stage. It is apparent that absence of ODAPH during the secretory stage did not impact the enamel rod pattern. This observation did not support the in vitro evidence that ODAPH is required as a nucleator of hydroxyapatite. Dental enamel formation may not require a nucleator (outside of what initiates dentin mineralization), as enamel mineral ribbons initiate on dentin mineral^41^ and are subsequently extended and oriented in close proximity to the ameloblast secretory surface^42–44^. Notably enamelin^45,46^ and ameloblastin^47^, but not amelogenin^43^, concentrate at the rod and interrod membrane growth sites and are absolutely necessary for enamel mineral ribbon initiation and elongation. The elongation of enamel mineral ribbons at a mineralization front along the ameloblast membrane is observed in all cases where true enamel forms, including humans^44^, mouse^48^, lungfish^48^, and gar^49^, and goes back to the origins of enamel during evolution.

Gar (*Lepisosteus*) is an actinopterygian, one of the most distant vertebrates from humans that makes enamel^50,51^. In the gar genome, only *Enam* and *Ambn* genes are found, whereas *Enam*, *Ambn*, and *Amel* are found in coelacanth^52^ and in tetrapods (both clades and lungfish constitute sarcopterygians) that make enamel, suggesting that *Amel* came into existence after the divergence of the common ancestor to gar and coelacanth and after the initial evolution of the enamel mineralization front. Similar to *Enam* and *Ambn*, *Odaph* is found in both sarcopterygians and actinopterygians, implying that *Odaph* is one of the most ancient enamel genes. Unlike mammalian *Odaph*, its non-mammalian orthologs, found in *Alligator*, lizard (*Anolis*), caecilian (*Geotryoetes*), gar, and reedfish (*Erpetoichthys*), consist of three exons and encode a Ser-Ser-Glu-Glu sequence (these two serine residues are potentially phosphorylated) and two or three cysteine residues (one in exon 2 and the other one or two in exon 3). Both introns are phase 1 (the intron is located between the first and the second nucleotides of a codon), confirming that *Odaph* is not a member of the SCPP gene family (all introns are located between two adjacent codons, phase 0). Mammalian *Odaph* genes consist of two exons, which is explained by the deletion of exon 2 in non-mammalian orthologs. One or two Cys and potentially phosphorylated Ser residues encoded by exon 3 of non-mammalian *Odaph* genes are common to most mammalian orthologs, including human *ODAPH*^23^.

Deviation from normal enamel formation in *Odaph*^C41*/C41*^ mice is evident at the end of ameloblasts' transition into maturation, when ameloblasts lose their columnar shape. A large cyst forms between the enamel and the ameloblasts, which flatten into a squamous morphology. The maturation stage protease *Klk4*, which is normally expressed by ameloblasts at the start of post-secretory transition^31,53–55^, is expressed by transition ameloblasts in *Odaph*^C41*/C41*^ mice and its expression continues even in the flattened ameloblasts, demonstrating that not all aspects of the transition had failed and that the flattened cells are indeed ameloblasts. In wild-type mice, it is evident during dissections that maturation ameloblasts are *firmly* attached to the enamel layer. Attempting to remove the enamel organ epithelia for the purpose of acquiring soft tissue samples for RT-PCR analyses, invariably results in the shredding of the enamel organ epithelia covering the enamel. This does not normally occur while collecting the soft-tissue covering secretory stage enamel. Consistent formation of a cyst at the onset of maturation in *Odaph*^C41*/C41*^ mice suggests that ODAPH plays a role in cell attachment directly, or indirectly by altering the expression of other genes necessary for attachment. It is also possible that failure of secreted ions to add to crystal surfaces could increase osmotic pressure that causes cyst formation.

Although secretory stage ameloblasts require the expression of the basement-membrane-associated genes *Col17a1*, *Lama3*, *Lamb3*, and *Lamc2*^56^, there is no visible basement membrane beneath secretory stage ameloblasts^57,58^. During transition and early maturation, in addition to the continued expression of laminin-332^59–61^, there is the added expression of a panel of novel SCPP genes that contribute to the attachment of maturation stage ameloblasts to the underlying enamel^62^. These genes include *Amtn*^63,64^, *Odam*^63^, *Scpppq1*^65^ and perhaps *Odaph*. Despite the expression of many basement membrane-derived genes, the attachment of ameloblasts to maturation stage enamel fails completely due of the absence of *Odaph*, a small, low expression, secreted phosphoprotein. Failure of the enamel layer to harden is the most severe that we have observed in any knockout exhibiting hypomaturation enamel specifically, and surpasses the hypomaturation defects displayed by the *Klk4*^39^ and *Wdr72*^40^ null mice.

The phylogenetic analysis indicates that *Odaph* evolved in reptiles and is present in monotremes, marsupials, and placental mammals. There are significant differences between the dental enamel formed in mammals and reptiles^66^. Mammalian enamel is generally thicker (posing a greater challenge for enamel maturation) and has a rod and interrod organization. The rod and interrod organization is the result of a special modification of the ameloblast distal membrane called the Tomes' process^67,68^ that elongates rod enamel mineral ribbons along the secretory surface of the protruding distal part of the process and interrod enamel mineral ribbons along the proximal part that encircles the cell and includes contributions from adjacent cells^69,70^. The rod/interrod organization of enamel is accomplished before *Odaph* is expressed, but such organization could alter the requirements of cell attachment during the maturation stage. KLK4, a protease that is expressed concurrently with ODAPH, degrades enamel proteins to facilitate their removal from the matrix, evolved even more recently^71^ than *Odaph* and is necessary for the maturation of dental enamel in most placental mammals.

## Supplementary Information

Supplementary Information.
