# Supplementary material for: Odontogenesis-associated phosphoprotein truncation blocks ameloblast transition into maturation in OdaphC41*/C41* mice
Source: Sci Rep. 2021 Jan 13;11:1132. doi: 10.1038/s41598-020-80912-y (PMC7807025; doi:10.1038/s41598-020-80912-y)
Supplement: Supplementary file 1 — Supplementary Information. [file 41598_2020_80912_MOESM1_ESM.docx]

Odontogenesis-Associated Phosphoprotein Truncation Blocks Ameloblast Transition into Maturation in *Odaph*^C41*/C41*^ Mice

**Authors:** Tian Liang^1^, Yuanyuan Hu^1^, Kazuhiko Kawasaki^3^, Hong Zhang^1^, Chuhua Zhang^1^, Thomas L. Saunders^2^, James P. Simmer^1^*, and Jan C-C. Hu^1^

**Affiliation:**

^1^Department of Biologic and Materials Sciences, University of Michigan School of Dentistry, 1210 Eisenhower Place, Ann Arbor, MI 48108, USA.

^2^Department of Internal Medicine, Division of Molecular, Medicine and Genetics, University of Michigan Medical School, Ann Arbor, MI 48109, USA.

^3^Department of Anthropology, Pennsylvania State University, University Park, Pennsylvania 16802.

**Supplemental figures:**

**Figure S1. CRISPR/Cas9 Strategy to Generate *Odaph*^C41*^ Knockout Mice.**

**Figure S2. *Odaph*^C41*^ Genotyping and Validation**.

**Figure S3. bSEM Images Normalized for Dentin Density Showing Relative Degrees of Mineralization.**


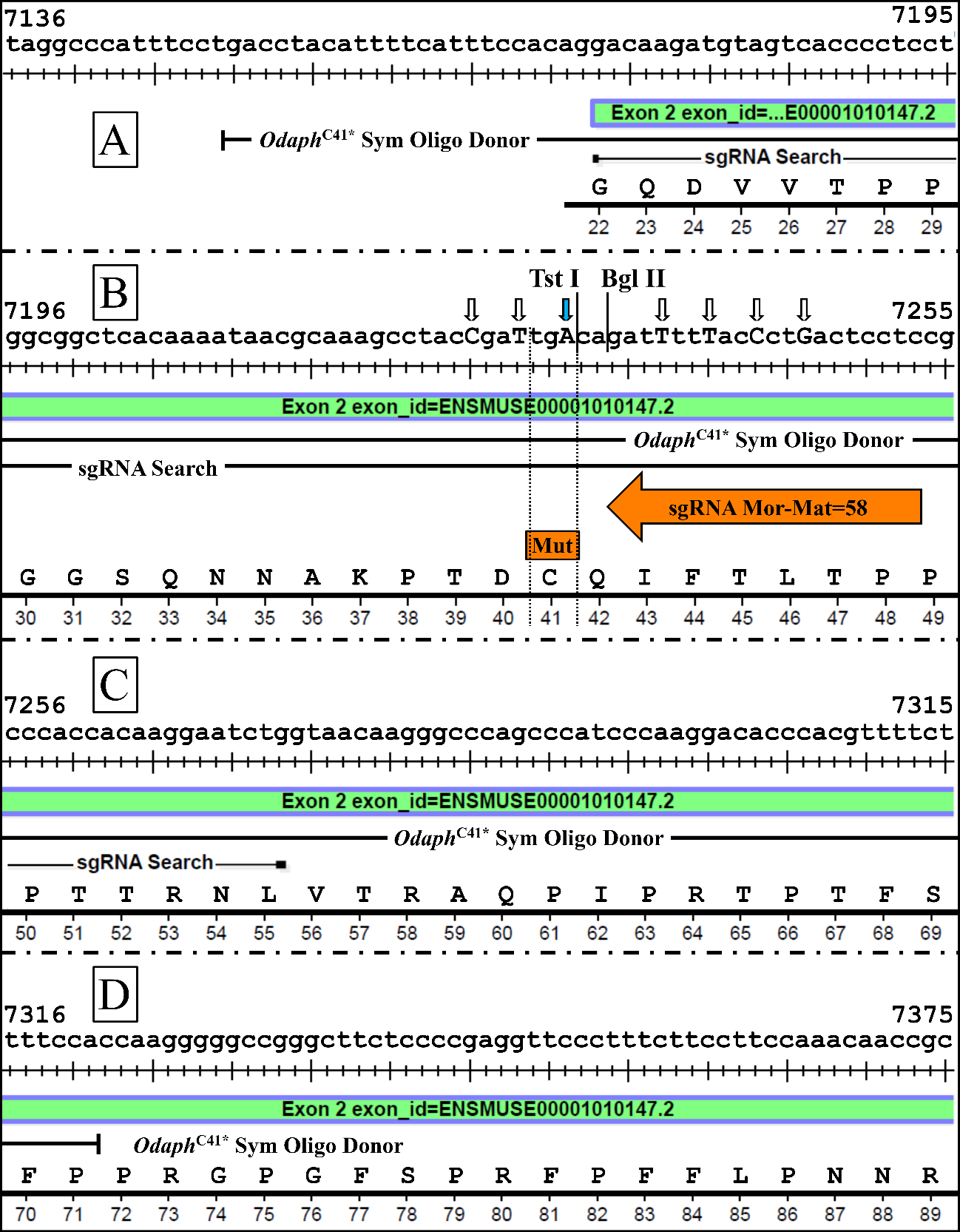


**Figure S1. CRISPR/Cas9 Strategy to Generate *Odaph*^C41*^ Knockout Mice.** The mouse C57BL/6J chromosome 5 region (91987473..91995317) containing the *Odaph* gene reference sequence (NC_000071.6) was analyzed to generate an *Odaph*^C41*^ mouse using CRISPR/Cas9 gene editing. The 5' region of *Odaph* exon2 (green bar) was analyzed to design the single guide RNA (sgRNA; orange arrow) to direct a Cas9 nuclease cleavage near the Cys41 codon. The symmetrical oligonucleotide donor (Sym Oligo Donor) provided a repair template guide facilitate homology-directed repair while introducing six silent variations (white arrows) and converted codon 41 into a TGA translation termination codon (blue arrow and orange box). These sequence variations facilitated later PCR genotyping by introducing a specific oligonucleotide priming site and eliminating a *Bgl*II (A'GATCT) restriction site.


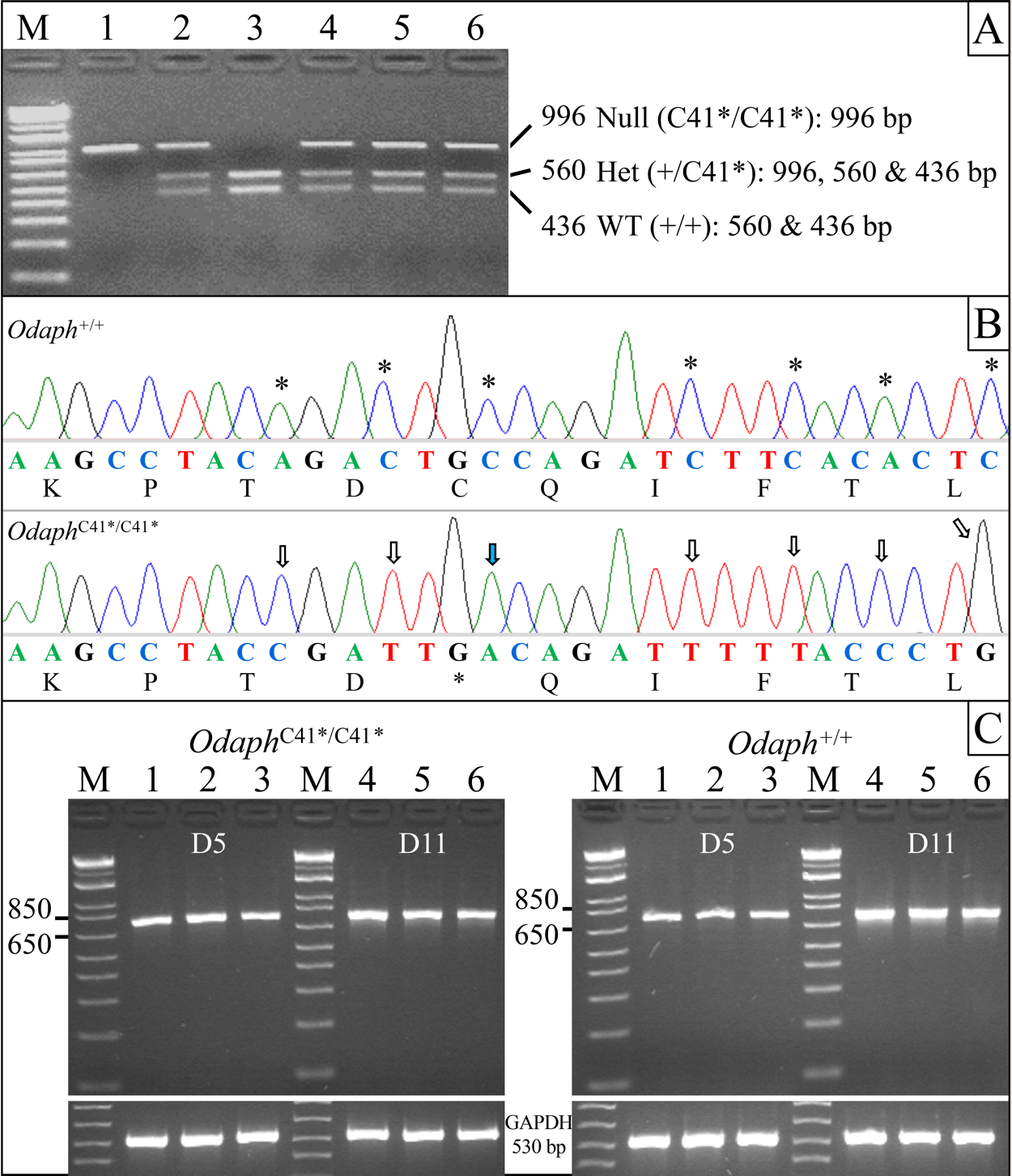


**Figure S2. *Odaph*^C41*^ Genotyping and Validation**. ***A:*** PCR amplified genomic DNA from tail biopsies is restricted with *BglII*, at the site eliminated by the CRISPR design. The *Odaph*^C41*^ amplification product, which is not cleaved by *BglII* digestion is 996 bp. The *Odaph*^+/+^ amplicon is restricted by *BglII* into 560 and 436 bp products. ***B:*** Genomic DNA from an *Odaph*^C41*/C41*^ mouse was amplified and characterized by DNA sequencing and showed no differences with the wild-type except those intended, which are shown in the DNA sequence chromatograms (arrows). ***C:*** RNA isolated from enamel organ epithelia (EOE) dissected from *Odaph*^C41*/C41*^ and *Odaph*^+/+^ first molars at D5 (secretory stage) and D11 (maturation stage) was amplified by RT-PCR. The *Odaph*^C41*^ allele appeared to be express normally in both stages of amelogenesis, and did not appear to undergo nonsense mediated decay. The 753 bp *Odaph*^C41*/C41^ amplification product was characterized by DNA sequencing and shown to have no sequence variations other than those introduced by design.


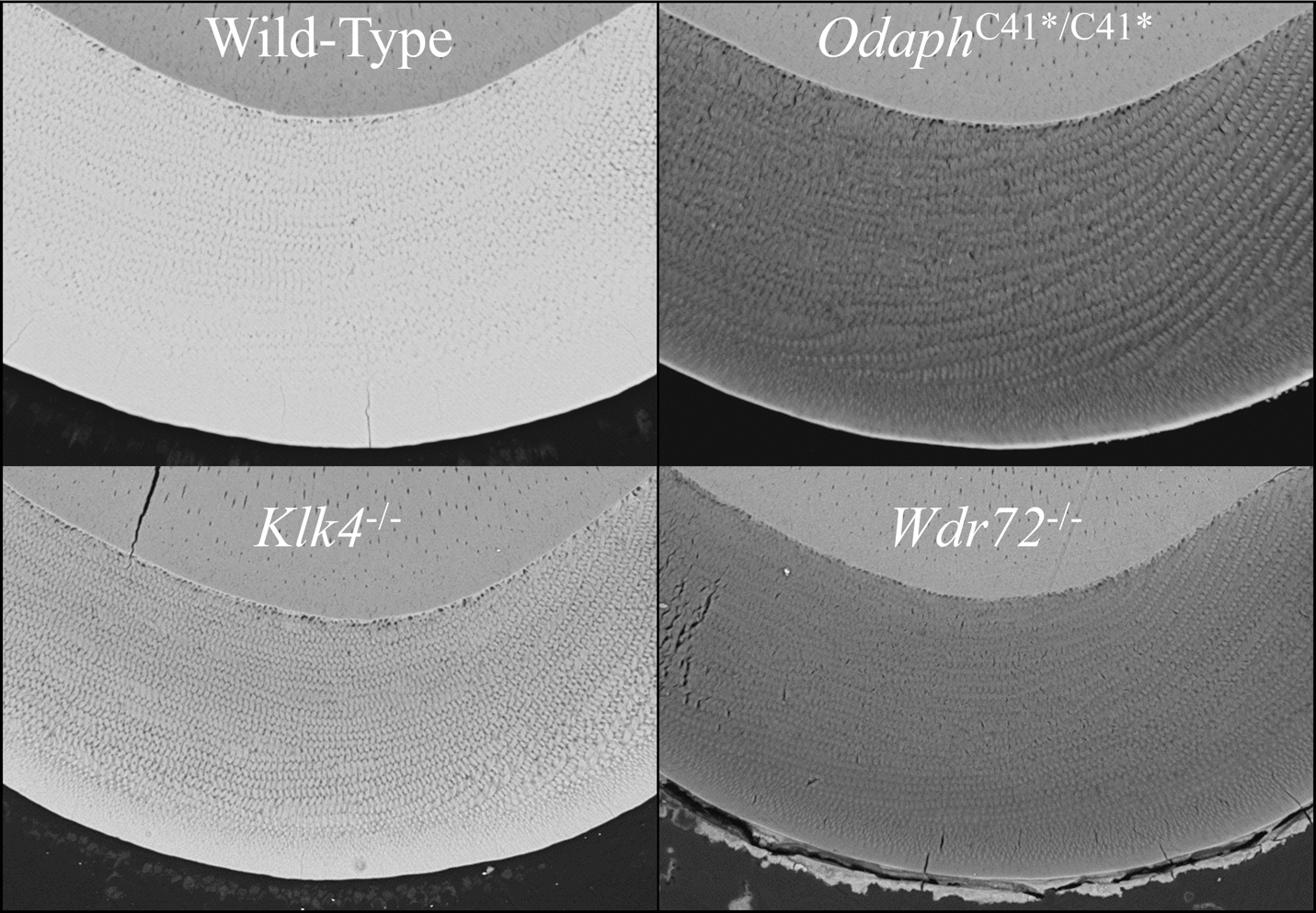


**Figure S3. bSEM Images Normalized for Dentin Density Showing Relative Degrees of Enamel Mineralization.** These images are taken from 7-week-old mandibular incisor cross-sections at Level 8, which is approximately at the level of the buccal crest of alveolar bone and immediately prior to its eruption into the oral cavity. In all four incisors the secretory stage proceeded normally, which established the full contour and thickness of the enamel layer as well as its rod and interrod architecture. Notice how the WT enamel is more highly mineralized than dentin (whiter). This is due to progressive hardening of the enamel after it was deposited during the secretory stage. The *Klk4*^-/-^ enamel is more highly mineralized on its surface and progressively less mineralized with depth. *Wdr72*^-/-^ enamel is poorly mineralized throughout and has an irregular crust on its surface. The *Odaph*^C41*/C41*^ incisor enamel is poorly mineralized throughout and is similar in density to the enamel layer at level 4, showing virtually no increase in density during the maturation stage.
